# Gender differences in metabolic syndrome and its components in southern china using a healthy lifestyle index: a cross-sectional study

**DOI:** 10.1186/s12889-023-15584-0

**Published:** 2023-04-12

**Authors:** Ying Ye, Qiuhong Zhou, Weiwei Dai, Hua Peng, Shi Zhou, Huixia Tian, Lu Shen, Huiwu Han

**Affiliations:** 1 Teaching and Research Section of Clinical Nursing, Xiangya Hospital of Central South University, Xiangya Road 87#, Changsha, Hunan P.R. China; 2 Xiangya School of Nursing, Central South University, Changsha, P.R. China; 3 National Clinical Research Center for Geriatric Disorders, Xiangya Hospital of Central South University, Changsha, Hunan P.R. China

**Keywords:** Metabolic health, Metabolic syndrome (MetS), Healthy lifestyle index (HLI), Gender, Risk factors

## Abstract

**Background:**

Lifestyle changes are important for the prevention and management of metabolic syndrome (MetS), but studies that focus on gender differences in the lifestyle risk factors of MetS are limited in China. This research aimed to generate a healthy lifestyle index (HLI) to assess the behavioral risk factors of MetS and its components, and to explore the gender differences in HLI score and other influencing factors of MetS.

**Methods:**

A convenience sample of 532 outpatients were recruited from a general hospital in Changsha, China. The general information and HLI scores [including physical activity (PA), diet, smoking, alcohol use, and body mass index (BMI)] of the subjects were collected through questionnaires, and each patient’s height, weight, waist circumference, and other physical signs were measured. Logistic regression analysis was used to analyze the risk factors of MetS and its components.

**Results:**

The prevalence of MetS was 33.3% for the whole sample (46.3% in males and 23.3% in females). The risk of MetS increased with age, smoking, unhealthy diet, and BMI in males and with age and BMI in females. Our logistic regression analysis showed that lower HLI (male: OR = 0.838,95%CI = 0.757–0.929; female: OR = 0.752, 95%CI = 0.645–0.876) and older age (male: OR = 2.899, 95%CI = 1.446–5.812; female: OR = 4.430, 95%CI = 1.640–11.969) were independent risk factors of MetS, for both sexes.

**Conclusion:**

Low levels of HLI and older ages were independent risk factors of MetS in both males and females. The association between aging and MetS risk was stronger in females, while the association between unhealthy lifestyles and MetS risk was stronger in males. Our findings reinforced the expected gender differences in MetS prevalence and its risk factors, which has implications for the future development of gender-specific MetS prevention and intervention programs.

**Supplementary Information:**

The online version contains supplementary material available at 10.1186/s12889-023-15584-0.

## Background

Metabolic syndrome (MetS) is a cluster of metabolic abnormalities including obesity, hyperglycemia (diabetes or impaired glucose regulation), hyperlipidemia (hypertriglyceridemia and/or low high-density lipoprotein cholesterolemia), and hypertension [1]. MetS has become a major global health issue, with a prevalence of 37.1% in the United States [2], 16.0% in Africa, 21.3% in Asia, 10.5% in Europe [3], over 21.5% in mainland China [4]. MetS seriously affects human health [1] and is associated with an increased risk of cardiovascular disease, type-2 diabetes, and all-cause mortality [5, 6].

MetS is the result of the interaction among social-environmental, behavioral, and physiological factors. Social-environmental factors include industrialization and urbanization [7], behavioral factors include lifestyle behaviors such as diet, physical activity, smoking, and alcohol use [8], and physiological factors include obesity, insulin resistance, hypertension, hyperlipidemia, and genetic susceptibility. Among the various influencing factors of MetS, lifestyle behaviors are the most modifiable factors that have been shown to play an essential role in the prevention and management of MetS among high-risk populations [9, 10].

Previous studies have identified five major lifestyle factors that were associated with MetS, including physical activity (PA), diet, tobacco use, alcohol use, and body mass index (BMI) [11, 12]. PA is a well-demonstrated protective factor to prevent and mitigate the impact of MetS [13]. Diet has also been found to be a key influencing factor of many chronic diseases including MetS. A diversified diet has been shown to help reduce obesity and improve metabolic health [14, 15]. Specifically, high-carbohydrate and low-fat diet has played an important role in the prevention and management of MetS in Asian populations [16, 17]. Tobacco use has also been associated with hyperlipidemia, hyperglycemia, and abdominal obesity [18, 19]. Specifically, smoking has been directly associated with an increased risk of developing MetS [20, 21]. In addition, both alcohol use and high BMI have been reported to be positively correlated with MetS [22]. Although the individual roles of these lifestyle factors have been extensively reported, little was known about their joint effects [23]. In order to better evaluate the overall lifestyle, the healthy lifestyle index (HLI) was created based on previous studies, which included five components: PA, diet, smoking, alcohol use, and BMI [11, 12].

Furthermore, amounting evidence has demonstrated significant gender differences in the prevalence of MetS in the United States, Europe, and Taiwan [24–26], with predominant evidence showing higher MetS prevalence in men than in women [24, 25]. In addition, studies have shown that men and women have different risk factors for MetS. For instance, men generally have higher rates of obesity and smoking, while women have higher rates of PA [27, 28]. A Chinese study found a higher risk of MetS in men who preferred an animal and fried food diet, and in women who preferred a high salt and energy diet [29]. Women prefer dairy products, vegetables, and fruits, while men prefer foods higher in salt and fat [28]. One study showed that as the educational level increased, the prevalence of MetS significantly decreased among women but increased among men [30]. A similar gender difference was also observed between employment and MetS risk, with a higher prevalence of MetS in white-collar men than blue-collar men, whereas the opposite was true in women [30]. Previous studies also showed significant gender differences in HLI [11, 23].

Previous studies using the HLI were mainly focused on patients with cancer [31, 32], hypertension [33], and type 2 diabetes [34], little is known about the association between HLI and MetS in the Chinese population, and even less is known about the gender differences in such an association. To fill the research gap, our study aimed to generate an HLI score that can be used to assess the behavioral risk factors of MetS and its components and to explore the gender differences in HLI score and other influencing factors of MetS.

## Methods

### Study design and setting

This cross-sectional study was carried out in a general Hospital in Changsha, China from March 2019 to December 2019. The STrengthening the Reporting of OBservational studies in Epidemiology (STROBE) checklist [35] was used in reporting this study (see Supplementary File 1).

### Study population

We used a convenience sampling method to select outpatients in the Cardiology Clinic, Endocrine Clinic, and Health Management Center of Xiangya Hospital, Central South University. Inclusion criteria were: (a) ≥ 18 years old; (b) able to read and write normally; (c) fully alert and conscious; and (d) with complete clinical data. Participants were excluded if they had the following conditions: (a) severe organ (heart, liver, or kidney) diseases; (b) other blood system or autoimmune diseases; (c) infectious diseases; (d) tumors; or (e) acute myocardial infarction, unstable angina pectoris, stroke, or acute cerebrovascular accidents within the last 6 months. Patients were recruited by nurses in our research group and were invited to complete a battery of paper-based questionnaires for data collection. Ethical approval was obtained from the ethics committee of the Hospital, and all study participants provided written informed consent.

The sample size was calculated by the formula for cross-sectional study [36]: *n* = Z_1-α/2_^2^ *p(1-p) /d^2^, where p (the overall prevalence of MetS in the Chinese population) was estimated at 31.1% based on a previous study [22], Z was set as 1.96 at a confidence interval of 95%, allowable error d was set as 5%, a sample size of 330 was required. Assuming a potential attrition rate of 15%, we finally recruited 594 subjects to the study, among who 62 were excluded due to missing data, leading to a final sample of 532 included in the statistical analysis.

### Measures

#### Definition of diseases

According to the latest Chinese guidelines [1], MetS refers to the presence of at least three of the following conditions: (a)Abdominal obesity: waist circumference(WC) ≥ 90 cm in men and ≥ 85 cm in women; (b) fasting blood glucose (FBG) ≥ 6.1 mmol/L or two-hour blood glucose ≥ 7.8 mmol/L after sugar loading or having ever been diagnosed and treated for diabetes; (c) blood pressure ≥ 130/85 mmHg or having ever been diagnosed and treated for hypertension; (d) fasting triglyceride (TG) ≥ 1.70 mmol/L; and(e) fasting high-density lipoprotein cholesterol < 1.04 mmol/L. In this study, hypertension was defined as systolic pressure ≥ 140 mmHg or diastolic pressure ≥ 90 mmHg; hyperglycemia was defined as fasting blood glucose ≥ 7 mmol/L; hyperlipidemia was defined as triglyceride(TG) > 2.3 mmol/L or total cholesterol(TC) > 6.2 mmol/L [37]; and abdominal obesity was defined as WC ≥ 90 cm in men and ≥ 85 cm in women, which was the same as the Chinese guidelines for MetS.

#### General information and assessment of patient lifestyles

We conducted face-to-face interviews to collect information on the participants’ socio-demographic characteristics (age, gender, education, and place of residence) and lifestyles (diet, smoking, alcohol use, sleep duration, and PA) using a self-designed structured questionnaire. The height, weight, and waist circumference (WC) of the participants were measured early in the morning after fasting. Height and weight were directly measured by a unified weighing scale with a ruler. WC was measured with a tape in the horizontal plane midway between the lowest rib and the iliac crest. BMI was calculated using the following formula: body weight [kg] / height-squared [m^2^]) [38].

Current smokers were defined as individuals who smoked > one cigarette/day, and former smokers were those who had ever smoked but had quit smoking. Participants were defined as never smokers if neither of the above cases was true [39]. Diet was evaluated by the intake of two types of beneficial food (vegetables & fruits, and beans) and two types of harmful food (pickled food, and sugary beverages) during the last week. Beneficial food intake was scored from 0 (never) to 7 (always), while harmful food was scored from 0 (always) to 7 (never). And a total diet score was the sum of the four types of food, which ranged from 0 to 28. We further categorized the total diet score into the following quintiles [40]: 0–5 points (1st quintile), 6–11 points (2nd quintile), 12–17 points(3rd quintile), 18–22 points (4th quintile), 23–28 points (5th quintile).

Participants’ levels of PA were evaluated using the Chinese version of the International Physical Activity Questionnaire-Short(IPAQ-S) [41]. The IPAQ-S consists of seven items that provide information on the time spent on high-intensity activities (such as aerobic exercise), moderate-intensity activities (such as leisure bicycling), and walking. Subjects were asked to recall the frequency and duration of each activity in the past seven days. The total PA score was calculated by multiplying the duration and frequency of PA with the metabolic equivalent (MET) value of each type of activity. We further divided the total PA score into three categories: low PA level, moderate PA level, and high PA level [42].

#### Definition of HLI

HLI was created based on previous studies [11] to assess five indicators related to a healthy lifestyle: PA, diet, smoking, alcohol use, and BMI. Each indicator was scored from 0–4 using the following criteria: PA (low = 0, moderate = 2, high = 4), diet (1st quintile = 0, 2nd quintile = 1, 3rd quintile = 2, 4th quintile = 3, 5th quintile = 4), smoking (current smoker = 0, former smoker = 2, never smoker = 4), alcohol use (> 60 g/day = 0, 24.0–59.9 g/day = 1, 12.0–23.9 g/day = 2, 6.0–11.9 g/day = 3, < 6.0 g/day = 4), BMI (≥ 30 = 0, 26–29.9 = 1, 24–25.9 = 2, 22–23.9 = 3, < 22 = 4). The total HLI score ranged from 0 to 20, with a higher score indicating a healthier lifestyle. According to previous studies [11, 43] and our results (nobody less than 5 points in the study), We further categorized the total HLI scores (**≤ **10, 11–15, ≥ 16) to describe them in more detail.

### Statistical analysis

All data analyses were conducted using SPSS 26.0. Incomplete data were not included in the final data analysis. Descriptive statistics were provided as medians and interquartile ranges for continuous variables, and numbers and percentages for categorical variables. The Mann–Whitney U test and chi-squared test were performed for group comparisons of continuous variables and categorical variables, respectively. Logistic regression was performed to explore the relationship between socio-demographic characteristics, HLI score, and behavioral risk factors for MetS and its components. A two-sided *p*-value < 0.05 was considered statistically significant.

## Results

### Characteristics of metabolic health and HLI

A total of 532 participants were included in the statistical analysis, of who 43.4% were males (*n* = 231) and 56.6% were females (*n* = 301). As shown in Table 1, the median (IQR) HLI score was 15 [13, 16] in the whole sample, 13 [11, 15] in males, and 15 [14, 17] in females. The prevalence of hypertension, hyperlipidemia, hyperglycemia, and abnormal WC in males was 38.1%, 32.0%, 12.2%, and 49.1%, respectively; while the prevalence of hypertension, hyperlipidemia, hyperglycemia, and abnormal WC in females was 25.0%, 25.9%, 12.2%, and 38.7%, respectively. The prevalence of MetS was 33.3% in the total sample, 46.3% in males, and 23.3% in females.

**Table 1:** Characteristics of metabolic diseases and HLI score (*n* = 532)

	**n (%)/ M(IQR)**	**Three categories of the HLI score n (%)**		
		** ≤ 10**	**11–15**	** ≥ 16**
**Male (***n* = 231**)**				
HLI score	13 (11,15)			
Hypertension	88 (38.1)	19 (21.6)	59 (67.0)	10 (11.4)
Hyperlipidemia	57 (32.0)	16 (28.1)	38 (66.7)	3 (5.3)
Hyperglycemia	22 (12.2)	0 (0)	21 (95.5)	1 (4.5)
Abnormal WC	113 (49.1)	19 (16.8)	72 (63.7)	22 (19.5)
MetS	107 (46.3)	27 (25.2)	69 (64.5)	11 (10.3)
**Female (***n* = 301**)**				
HLI score	15 (14,17)			
Hypertension	75 (25.0)	0 (0)	45 (60.0)	30 (40.0)
Hyperlipidemia	57 (25.9)	1 (1.8)	33 (57.9)	23 (40.4)
Hyperglycemia	27 (12.2)	2 (7.4)	15 (55.6)	10 (37.0)
Abnormal WC	116 (38.7)	2 (1.7)	64 (55.2)	50 (43.1)
MetS	70 (23.3)	3 (4.3)	47 (67.1)	20 (28.6)

*Abbreviations*: *HLI* healthy lifestyle index, *WC* waist circumference, *MetS* Metabolic syndrome, *IQR* interquartile range

### HLI score and social demographics by MetS syndromes

Table 2 presents the comparisons of HLI scores and socio-demographic characteristics by various metabolic syndromes in both genders. As for males, compared to non-MetS patients, MetS patients were older (78.5% vs. 21.5%), less likely to have a diet score of 2nd quintile (12.1% vs 25.8%) and 3rd quintile (54.2% vs 56.5%), more likely to have a smoking history (61.6% vs. 46.8%) and with BMI ≥ 26 kg/m^2^ (57% vs.16.9%). In addition, non-MetS patients had a higher HLI total score than MetS patients [14(12,16) vs 13(10,14)]. For females, compared to non-MetS patients, MetS patients were older (90.0% vs. 10.0%), had lower education (senior high school and below: 87.1% vs. 50.2%), were more like to live in rural areas (54.3% vs. 45.7%), sleep < 6 h (45.7% vs 19.9%), and with BMI ≥ 24 kg/m^2^ (75.7% vs. 26.5%). Moreover, non-MetS patients had a higher HLI total score than MetS patients [16(14,17)vs14(13,16)].

**Table2:** Comparisons of HLI scores and socio-demographic characteristics by various metabolic syndromes (*n* = 532)

	**Hypertension**		**Hyperlipoidemia**		**Hyperglycemia**		**Abdominal obesity**		**Metabolic syndrome**	
	**Yes**	**No**	**Yes**	**No**	**Yes**	**No**	**Yes**	**No**	**Yes**	**No**
**Male *n* = 231**										
Diet score n(%)										
1st quintile	0	0	0	0	0**	0	0	0	0**	0
2nd quintile	32(22.4)	13(14.8)	14(24.6)	21(17.4)	0	36(22.6)	22(19.5)	22(18.8)	13(12.1)	32(25.8)
3rd quintile	80(55.4)	48(54.5)	30(52.6)	68(56.2)	11(50.0)	86(54.1)	64(56.6)	64(54.7)	58(54.2)	70(56.5)
4th quintile	31(21.7)	27(30.7)	13(28.9)	32(26.4)	11(50.0)	37(23.3)	27(23.9)	31(26.5)	36(33.6)	22(17.7)
5th quintile	0	0	0	0	0	0	0	0	0	0
Alcohol (g/d) n(%)										
< 6	82(93.2)	139(97.2)	55(96.5)	116(95.9)	22(100.0)	151(95.0)	107(94.7)	113(96.6)	101(94.4)	120(96.8)
6–11.9	6(6.8)	2(1.4)	1(1.8)	5(4.1)	0	7(4.4)	5(4.4)	3(2.6)	5(4.7)	3(2.4)
12–24.9	0	1(0.7)	1(1.8)	0	0	1(0.6)	1(0.9)	0	1(0.9)	0
25–59.9	0	1(0.7)	0	0	0	0	0	1(0.9)	0	1(0.8)
> 60	0	0	0	0	0	0	0	0	0	0
PA intensity n(%)							*			
Low	29(33.0)	50(35.0)	23(40.4)	41(33.9)	12(54.5)	52(32.7)	30(26.5)	48(41.0)	44(41.1)	35(28.2)
Moderate	51(58.0)	72(50.3)	27(47.4)	65(53.7)	7(31.8)	86(54.1)	70(61.9)	53(45.3)	51(47.7)	72(58.1)
High	8(9.1)	21(14.7)	7(12.3)	15(12.4)	3(13.6)	21(13.2)	13(11.5)	16(13.7)	12(11.2)	17(58.6)
BMI (kg/m^2^) n(%)	**		**				*		**	
< 22	12(13.6)	31(21.7)	2(3.5)	26(21.5)	2(9.1)	27(17.0)	14(12.4)	29(24.8)	7(6.5)	36(29.0)
22–23.9	17(19.3)	38(26.6)	10(17.5)	34(28.1)	5(22.7)	40(25.2)	31(27.4)	23(19.7)	22(20.6)	33(26.6)
24–25.9	15(17.0)	36(25.2)	12(21.1)	27(22.3)	4(18.2)	34(21.4)	28(24.8)	23(19.7)	17(15.9)	34(27.4)
26-29.9	36(40.9)	35(24.5)	27(47.4)	33(27.3)	11(50.0)	52(32.7)	32(28.3)	39(33.3)	51(47.7)	20(16.1)
≥ 30	8(9.1)	3(2.1)	6(10.5)	1(0.8)	0	6(3.8)	8(7.1)	3(2.6)	10(9.3)	1(0.8)
Smoking n(%)	**								**	
Current	27(30.7)	54(37.8)	23(40.4)	48(39.7)	4(18.2)	65(40.9)	37(32.7)	44(37.6)	36(33.6)	45(36.3)
Former	29(33.0)	14(9.8)	9(15.8)	22(18.2)	6(27.3)	28(17.6)	25(22.1)	18(15.4)	30(28.0)	13(10.5)
Never	32(36.4)	75(52.4)	25(43.9)	51(42.1)	12(54.5)	66(41.5)	51(45.1)	55(47.0)	41(38.3)	66(53.2)
HLI total score M(IQR)	13(11,15)	13(11,16)	* 13(10,14)	13(11,15)	13(12,14)	13(11,15)	13(11,15)	13(11,15)	** 13(10,14)	14(12,16)
Age n(%)									**	
< 45	23(26.1)	50(35.0)	19(33.3)	36(29.8)	4(18.2)	53(33.3)	31(27.4)	42(35.9)	23(21.5)	50(40.3)
≥ 45	65(73.9)	93(65.0)	38(66.7)	85(70.2)	18(81.8)	106(66.7)	82(72.6)	75(64.1)	84(78.5)	74(59.7)
Education n(%)	**									
Senior high school and below	54(61.4)	74(51.7)	27(47.4)	72(59.5)	11(50.0)	87(54.7)	66(58.4)	62(53.0)	61(57.0)	67(54.0)
College degree / Undergraduate	34(38.6)	57(39.9)	25(43.9)	42(34.7)	11(50.0)	60(37.7)	42(37.2)	48(41.0)	43(40.2)	48(38.7)
Graduate degree and above	0	12(8.4)	5(8.8)	7(5.8)	0	12(7.5)	5(4.4)	7(6.0)	3(2.8)	9(7.3)
Place of residence (%)										
Urban	56(63.6)	85(59.9)	35(61.4)	74(61.7)	16(72.7)	97(61.4)	66(58.9)	74(63.2)	66(62.3)	75(60.5)
Rural	32(36.4)	57(40.1)	22(38.6)	46(38.3)	6(27.3)	61(38.6)	46(41.1)	43(36.8)	40(37.7)	49(39.5)
Sleep length/day n (%)										
< 6 h	15(17.0)	32(22.4)	11(19.3)	29(24.0)	8(36.4)	32(20.1)	20(17.7)	27(23.1)	24(22.4)	23(18.5)
6–8 h	56(63.6)	85(59.4)	38(66.7)	71(58.7)	11(50.0)	100(62.9)	72(63.7)	68(58.1)	66(61.7)	75(60.5)
> 8 h	17(19.3)	26(18.2)	8(14.0)	21(17.4)	3(13.6)	27(17.0)	21(18.6)	22(18.8)	17(15.9)	26(21.0)
	**Hypertension**		**Hyperlipidemia**		**Hyperglycemia**		**Abdominal obesity**		**Metabolic syndrome**	
	**Yes**	**No**	**Yes**	**No**	**Yes**	**No**	**Yes**	**No**	**Yes**	**No**
**Female *n* = 301**										
Diet score n(%)										
1st quintile	0	0	0	0	0	0	0	0	0	0
2nd quintile	1(1.3)	2(0.9)	2(1.2)	1(1.8)	3(1.5)	0	3(1.6)	0	0	3(1.3)
3rd quintile	12(16.0)	29(12.9)	26(16.0)	8(23.5)	30(15.5)	1(3.2)	22(12.0)	19(16.4)	9(12.9)	32(13.9)
4th quintile	47(62.7)	141(62.7)	102(62.6)	34(59.6)	120(61.9)	18(66.7)	113(61.4)	75(64.7)	44(62.9)	144(62.6)
5th quintile	15(20.0)	53(23.6)	33(20.2)	14(29.8)	41(21.1)	8(29.6)	46(25.0)	22(19.0)	17(24.3)	51(22.2)
Alcohol (g/d) n(%)										
< 6	75(100.0)	224(99.1)	57(100.0)	162(99.4)	27(100.0)	194(99.5)	116(99.1)	183(99.5)	70(100.0)	229(99.1)
6–11.9	0	1(0.4)	0	0	0	0	1(0.9)	0	0	1(0.4)
12–24.9	0	0	0	0	0	0	0	0	0	0
25–59.9	0	1(0.4)	0	1(0.6)	0	1(0.5)	0	1(0.5)	0	1(0.4)
> 60	0	0	0	0	0	0	0	0	0	0
PA intensity n(%)										
Low	28(37.3)	83(36.7)	24(42.1)	55(33.7)	9(33.3)	71(36.4)	44(37.6)	67(36.4)	28(40.0)	83(35.9)
Moderate	32(42.7)	117(51.8)	26(45.6)	84(51.5)	15(55.6)	97(49.7)	58(49.6)	91(49.5)	30(42.9)	119(51.5)
High	15(20.0)	26(11.5)	7(12.3)	24(14.7)	3(11.1)	27(13.8)	15(12.8)	26(14.1)	12(17.1)	29(12.6)
BMI (kg/m^2^) n(%)	**		**						**	
< 22	20(26.7)	111(49.1)	12(21.1)	83(50.9)	6(22.2)	89(45.6)	44(37.6)	87(47.3)	6(8.6)	125(54.1)
22–23.9	19(25.3)	37(16.4)	19(33.3)	21(12.9)	6(22.2)	32(16.4)	22(18.8)	34(18.5)	11(15.7)	45(19.5)
24–25.9	13(17.3)	38(16.8)	12(21.1)	27(16.6)	6(22.2)	35(17.9)	24(20.5)	27(14.7)	21(30.0)	30(13.0)
26–29.9	16(21.3)	29(12.8)	11(19.3)	22(13.5)	7(25.9)	28(14.4)	18(15.4)	27(14.7)	22(31.4)	23(10.0)
≥ 30	7(9.3)	11(4.9)	3(5.3)	10(6.1)	2(7.4)	11(5.6)	9(7.7)	9(4.9)	10(14.3)	8(3.5)
Smoking n(%)										
Current	1(1.3)	4(1.8)	1(1.8)	3(1.8)	2(7.4)	2(1.0)	3(2.6)	2(1.1)	3(4.3)	2(0.9)
Former	0	0	0	0	0	0	0	0	0	0
Never	74(98.7)	222(98.2)	56(98.2)	160(98.2)	25(92.6)	193(99.0)	114(97.4)	182(98.9)	67(95.7)	229(99.1)
HLI total score M(IQR)	15(13,17)	15(14,17)	15(14,16)	15(14,17)	15(14,17)	15(14,17)	15(14,17)	15(14,17)	** 14(13,16)	16(14,17)
Age n(%)	**		**		**		**		**	
< 45	9(12.0)	123(54.4)	16(28.1)	81(49.7)	5(18.5)	95(48.7)	40(34.2)	92(50.0)	7(10.0)	125(54.1)
≥ 45	66(88.0)	103(45.6)	41(71.9)	82(50.3)	22(81.5)	100(51.3)	77(65.8)	92(50.0)	63(90.0)	106(45.9)
Education n(%)	**		**		**				**	
Senior high school and below	66(88.0)	111(49.1)	42(73.7)	81(49.7)	23(85.2)	120(52.3)	77(65.8)	100(54.3)	61(87.1)	116(50.2)
College degree / Undergraduate	9(12.0)	98(43.4)	13(22.8)	70(42.9)	4(14.8)	79(40.5)	36(30.8)	71(38.6)	9(12.9)	98(42.4)
Graduate degree and above	0	17(7.5)	2(3.5)	12(7.4)	0	14(7.2)	4(3.4)	13(7.1)	0	17(7.4)
Place of residence(%)	**								**	
Urban	38(50.7)	159(70.4)	36(63.2)	112(68.7)	14(51.9)	137(70.3)	69(59.0)	128(69.6)	32(45.7)	165(71.4)
Rural	37(49.3)	67(29.6)	21(36.8)	51(31.3)	13(48.1)	58(29.7)	48(41.0)	56(30.4)	38(54.3)	66(28.6)
Sleep length/day n (%)	**						*		**	
< 6 h	34(45.3)	44(19.5)	13(22.8)	40(24.5)	10(37.0)	44(22.6)	39(33.3)	39(21.2)	32(45.7)	46(19.9)
6–8 h	34(45.3)	153(67.7)	37(64.9)	107(65.6)	13(48.1)	131(67.2)	61(52.1)	126(68.5)	30(42.9)	157(68.0)
>8h	7(9.3)	29(12.8)	7(12.3)	16(9.8)	4(14.8)	20(10.3)	17(14.5)	19(10.3)	8(11.4)	28(12.1)

*Abbreviations*: *PA* physical activity, *BMI* body mass index, *HLI* healthy lifestyle index, *IQR* interquartile range

^*^Denotes *P* < 0.05

^**^Denotes *P* < 0.01

### Influencing factors of MetS

The results of the logistic regression analyses of MetS influencing factors in males and females are summarized in Table 3. All significant variables in the univariate analysis and possible confounding factors were included in the regression analysis. The results showed that both HLI score and age were significant influencing factors of MetS in both genders. A higher HLI score was associated with a lower risk of MetS in both males (OR:0.838; 95%CI:0.757–0.929) and females (OR: 0.752, 95%CI: 0.645–0.876). In addition, older age was associated with a higher risk of MetS in both males (OR:2.899, 95%CI: 1.446–5.812) and females (OR:4.430; 95%CI: 1.640–11.969).

**Table3:** Logistic regression analysis of MetS risk factors (*n* = 532)

Variables	OR	95%CI	*P* value
**Male *n* = 231**			
HLI total	0.838	0.757–0.929	0.001
Age(years)			
< 45^a^	1.000		
≥ 45	2.899	1.446–5.812	0.003
Education			
Senior high school and below ^a^	1.000		
College degree/Undergraduate	1.264	0.614–2.602	0.525
Graduate degree and above	0.679	0.147–3.128	0.619
Place of residence			
Rural ^a^	1.000		
Urban	1.110	0.570–2.161	0.760
Sleep length/day			
< 6 h ^a^	1.000		
6–8 h	0.968	0.479–1.953	0.927
> 8 h	0.539	0.221–1.312	0.173
**Female *n* = 301**			
HLI total	0.752	0.645–0.876	< 0.001
Age(years)			
< 45 ^a^	1.000		
≥ 45	4.430	1.640–11.969	0.003
Education			
Senior high school and below ^a^	1.000		
College degree/Undergraduate	0.551	0.208–1.459	0.231
Graduate degree and above	0.000	0.000	0.998
Place of residence			
Rural ^a^	1.000		
Urban	0.755	0.393–1.451	0.399
Sleep length/day			
< 6 h ^a^	1.000		
6–8 h	0.555	0.284–1.088	0.086
> 8 h	0.601	0.215–1.678	0.331

*Abbreviations*: *HLI* healthy lifestyle index, *MetS* Metabolic syndrome

^a^ Denotes reference category

## Discussion

This study explored the gender differences in behavioral risk factors of MetS. Our study showed a significantly higher prevalence of hypertension, hyperlipidemia, abdominal obesity, and MetS in males than in females. However, some other studies have found that the prevalence of MetS was higher in females than in males [44, 45]. The different results from different studies may be explained by the differences in hormones, lipid metabolism, population diversity, cultural behaviors, lifestyle habits, and the use of different diagnostic criteria. Some research has found that gender may be an independent predictor of most MetS components. Although hypertension, pre-diabetes, and hypertriglyceridemia are more common in males than in females, the prevalence of low high-density lipoprotein and high WC is higher in females than in males [46].

Our results showed that both HLI and age were independent influencing factors for MetS, for both sexes. What’s more, the association between age and MetS was stronger in females, which may be explained by the redistribution of fat due to decreased estrogen levels during menopause among females [38]. On the other hand, the association between lifestyle and MetS was stronger in males, which may be due to poor compliance with healthy lifestyles among males further affecting their metabolic health.

A healthy lifestyle has been well-established to play a critical role in preventing or delaying the occurrence of MetS [47]. In general, our study showed that women had healthier lifestyles than men, while men were more likely to engage in unhealthy behaviors such as smoking, alcohol use, and unhealthy diets than women, which is consistent with previous studies [28]. In addition, our study showed that the prevalence of abdominal obesity was higher in males than in females, which was consistent with previous findings and may be explained by the healthier life habits of females than males [30]. In terms of the five components of HLI, higher BMI was associated with a higher risk of MetS in both males and females, which was consistent with previous studies [22, 48]. Kobo et al.’s cohort study showed that people with higher BMI were more likely to develop MetS, while normal BMI can rule out MetS [49]. In addition, smoking and unhealthy diets were influencing factors of MetS in males. In 2019, there were 341 million smokers in China, of which 93.3% were males, and smoking has been widely demonstrated to increase the risk of MetS [18, 50]. Studies have shown that the prevalence of MetS increased in individuals with unhealthy dietary habits [51]. Healthy dietary patterns such as the Mediterranean diet [52] and diets rich in Omega-3 fatty acids [53] can decrease the risk of MetS. These findings suggest that multiple measures may be effective in preventing and reducing the risk of MetS in males and females, such as reducing BMI, avoiding smoking and alcohol use, and maintaining healthy diets.

Our study showed that the prevalence of MetS increased gradually with age, with a significantly higher prevalence of MetS in the ≥ 45 years old age group than the < 45 years old age group. This finding was consistent with previous studies and further corroborated age as the most important determinant of cardiovascular risk [54, 55]. Additionally, older patients had more metabolic syndromes than younger patients [56]. In particular, one previous study showed that the prevalence of MetS peaked at age 40–59 in males, but at a later age of ≥ 60 in females [57]. The increased risk of MetS with older age may be explained by multiple factors, including the accumulation of risk factors, the hormonal changes (especially in women), and the decreasing secretory function of pancreatic β cells over time, which all increase the risk of insulin resistance and hence the risk of MetS [58].

Our study was one of the few studies that focused on gender differences in lifestyle risk factors for MetS. We generated a healthy lifestyle index (HLI) to assess the overall behavioral risk factors of metabolic syndrome and its components and explored the gender differences in HLI score and other influencing factors of MetS. However, there are some limitations to this study. First, the cross-sectional study design may preclude any causal inference between MetS and its influencing factors. Second, the sample was recruited from one hospital and may lack representation. Third, the data were all collected by non-validated questionnaire and based on self-report, which may be subject to bias. Fourth, the assessment of dietary habits was limited to certain kinds of food and may not be comprehensive enough. Finally, we didn’t include psychological factors in our exploration of MetS influencing factors, though previous evidence has shown that psychological stress was related to an increased risk of MetS [59].

## Conclusion

Our study showed a higher prevalence of MetS in males than females. In addition, low levels of HLI and older ages were independent risk factors of MetS in both males and females. The association between aging and MetS risk was stronger in females, while the association between unhealthy lifestyles and MetS risk was stronger in males. Our findings reinforced the expected gender differences in MetS prevalence and its risk factors, which provides important guidance for the future development of gender-specific prevention and intervention programs to prevent and reduce the risk of MetS in both males and females. At a political level, a healthy lifestyle should be listed as a national population health plan to encourage people to develop and maintain healthy habits to prevent MetS. At an individual level, males and females may have different focuses in the prevention of MetS. For instance, males may focus on changing unhealthy lifestyle habits, such as smoking, alcohol use, and unhealthy diet, while females may focus on the impact of hormone levels after menopause.

## Supplementary Information

**Additional file 1.**

## Data Availability

The data sets used and/or analyzed during the current study are available from the corresponding author upon reasonable request.

## Abbreviations

MetS: Metabolic syndrome
HLI: Healthy lifestyle index
WC: Waist circumference
IQR: Interquartile rang
PA: Physical activity
BMI: Body mass index
FBG: Fasting blood glucose
TG: Triglyceride
TC: Total cholesterol
IPAQ-S: International Physical Activity Questionnaire-Short
MET: Metabolic equivalent
